# Effect of Repeated Bolus and Continuous Glucose Infusion on DNA Damage and Oxidative Stress Biomarkers in Healthy Male Volunteers

**DOI:** 10.3390/ijms241713608

**Published:** 2023-09-02

**Authors:** Laura Bragagna, Christina Polak, Lisa Schütz, Lina Maqboul, Carmen Klammer, Roland Feldbauer, Agnes Draxler, Martin Clodi, Karl-Heinz Wagner

**Affiliations:** 1 Department of Nutritional Sciences, University of Vienna, 1090 Vienna, Austria; laura.bragagna@univie.ac.at (L.B.); lina.maqboul@univie.ac.at (L.M.); agnes.draxler@univie.ac.at (A.D.); 2 Vienna Doctoral School of Pharmaceutical, Nutritional and Sport Sciences, University of Vienna, 1090 Vienna, Austria; carmen.klammer@bblinz.at; 3 Department of Internal Medicine, St. John of God Hospital Linz, 4020 Linz, Austria; roland.feldbauer@bblinz.at (R.F.); martin.clodi@bblinz.at (M.C.); 4 ICMR–Institute for Cardiovascular and Metabolic Research, Johannes Kepler University Linz (JKU Linz), 4040 Linz, Austria

**Keywords:** glucose variability, glucose, type 2 diabetes, oxidative stress, hyperglycemia, DNA damage, comet assay, protein carbonyls, bilirubin, antioxidants, ROS

## Abstract

Glucose variability (GV), which describes fluctuations in blood glucose levels within the day, is a phenomenon that is increasingly becoming the target of scientific attention when it comes to increased risk of coronary heart disease. Effects of GV may contribute to the development of metabolic syndrome and type 2 diabetes. Hyperglycemia can lead to oxidative stress resulting in molecular damage due to accumulation of reactive oxygen species (ROS). To discover more about the immediate effects of GV, continuous vs. bolus intravenous glucose administration was applied to 10 healthy men aged 21–30 years over a time frame of 48 h. Whole blood and plasma were analyzed for DNA damage using a comet assay with 3 different treatments (lysis buffer, H_2_O_2_, and the lesion-specific enzyme formamidopyrimidine DNA glycosylase (FPG)) as well as for the oxidative stress markers protein carbonyls (PC), unconjugated bilirubin (UCB), and ferric reducing antioxidant power (FRAP). A significant time effect was found in the three DNA damage treatments as well as in PC and UCB possibly due to circadian changes on oxidative stress, but no intervention group effect was observed for any of the markers. In conclusion, bolus vs. continuous glucose administration had no significant acute effect on DNA damage and markers of oxidative stress in healthy men.

## 1. Introduction

Type 2 diabetes, with about 400 million global cases, is a widespread disease with the characteristic of hyperglycemia, which leads to a two-fold higher risk of vascular diseases and an increased risk of mortality [1,2]. While the effects of type 2 diabetes and thereby hyperglycemia on secondary diseases have already been described in numerous studies, glucose variability (GV) is a topic that is comparatively new. GV describes the phenomenon of fluctuations of within-day blood glucose levels, which includes phases of hypo- and hyperglycemia [3]. GV is also found in individuals with normal glucose tolerance; however, it is increased in individuals with type 2 diabetes and impaired glucose regulation [4]. The potential implications of GV on type 2 diabetes and possibly related diseases have not been adequately investigated. Oxidative stress is a state that occurs with an imbalance between the production and elimination of reactive oxygen and nitrogen species (ROS, RNS) [5]. Experiments with cultured aortic endothelial cells have already demonstrated how hyperglycemia can induce ROS accumulation through the overproduction of superoxide (SO) by the mitochondrial electron-transport chain. Due to an overproduction of electron donors by the tricarboxylic acid (TCA) cycle, hyperglycemia increases the proton gradient at the inner mitochondrial membrane and subsequently leads to an increase in the electrochemical potential difference above a threshold that prolongs the lifetime of SO-generating electron transport intermediates such as ubisemiquinone. This accumulation of SO further leads to changes in metabolic pathways such as an increased polyol pathway flux, increased formation of advanced glycation end products (AGEs), activation of protein kinase C (PKC), and increased flux through the hexosamine pathway. These changes in metabolic pathways further lead to oxidative stress, which induces oxidative damage in DNA, lipids, and proteins. In the long term, oxidative stress can lead to harmful effects to cellular structures and subsequently organ damage and a variety of diseases, especially diabetic complications such as diabetic cardiomyopathy and micro- and macrovascular diseases [6,7,8,9,10,11]. Therefore, in type 2 diabetes patients, increased oxidative damage or lower antioxidant levels could be assessed [12,13,14], although these values vary depending on good medical treatment [15,16]. Based on these observations, more information is needed on the influence of GV on oxidative stress markers. The purpose of this study was to contribute to a better understanding of short-term effects of GV, in particular, bolus vs. continuous intravenous administrations of glucose in the time span of 48 h, on DNA damage and oxidative stress in the blood of healthy volunteers. While long-term hyperglycemia is usually measured by the percentage of glycated hemoglobin HbA1c, it is not suitable for measuring short-term GV. Continuous glucose monitoring (CGM) was therefore the applied method for this purpose [17]. Due to the relatively short measurement period, it was also reasonable to focus on oxidative stress markers, which respond sensitively to short-term changes in the body. Those markers included DNA damage, the ferric reducing antioxidant power (FRAP), unconjugated bilirubin (UCB), and protein carbonyls (PCs). A special focus in this study was given to DNA damage as studies showed increased DNA damage in different cell lines at elevated glucose concentrations [18,19,20,21]. The method of choice to determine DNA damage was the well-established comet assay, which allows specific determination of single- and double-strand breaks in the DNA of individual cells. While the standard protocol provides gel electrophoresis after lysis of the cells, an additional treatment with hydrogen peroxide (H_2_O_2_) allows an indirect measurement of the antioxidant status in cells and reflects the resistance to H_2_O_2_. In addition, a treatment with the lesion-specific enzyme formamidopyrimidine DNA glycosylase (FPG) was used to detect oxidized purines, mainly 8-oxoguanine.

## 2. Results

### 2.1. Baseline Characteristics

As described earlier by Feldbauer et al. [22], 10 male volunteers with a mean age of 25 ± 3 (mean ± standard deviation) years (Min 21/Max 30) and a mean BMI of 25.6 ± 2.5 kg/m^2^ (22.5/30.9) participated in the study. The mean waist circumference was 90 ± 8 cm (81/110), and the resting heart rate of the volunteers mean was 62 ± 11 bpm (53/88). The characteristics of the subjects are described in Table 1.

### 2.2. Continuous Glucose Monitoring

**Figure 1:**
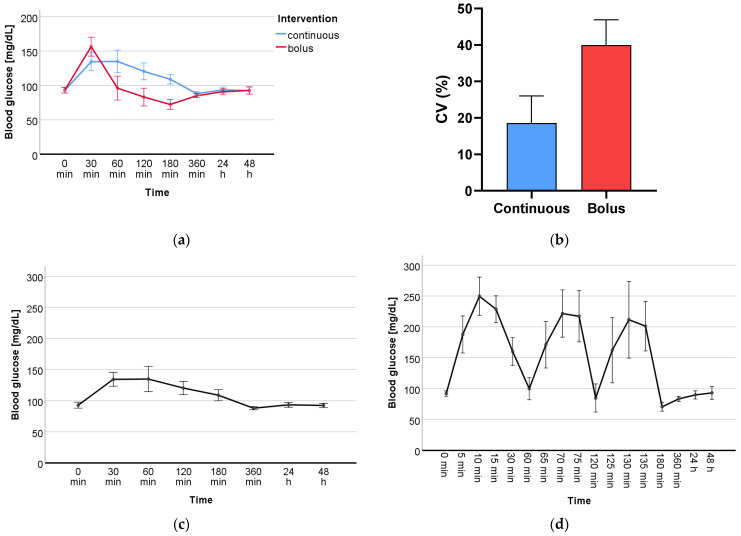
(**a**) Levels of blood glucose (mg/dL) of continuous vs. bolus over the course of the 8 time points where blood was drawn. (**b**) Mean value of the coefficient of variation (CV) (%) of the two intervention days, continuous and bolus. Isolated presentation of the progression of blood glucose (mg/dL) over all measured time points during (**c**) continuous and (**d**) bolus glucose administration.

Blood glucose levels (mg/dL) were determined on both study days (continuous and bolus administration) at 8 time points (0 min, 30 min, 60 min, 120 min, 180 min, 360 min, 24 h, 48 h), at which time also blood and plasma were collected for oxidative stress marker analyses. During bolus administration, blood glucose was also determined at 9 additional time points (5 min, 10 min, 15 min, 65 min, 70 min, 75 min, 125 min, 130 min, 135 min) in order to detect fluctuations due to rapid glucose administration in more detail. Blood glucose levels of the two measurement days are shown in Table A1. Repeated-measures analysis of variance (RM-ANOVA) revealed a statistically significant difference (*p* < 0.001) between the blood glucose levels of the two groups, continuous and bolus, and over the course of the 8 time points (*p* < 0.001), as shown visually in Figure 1a. The coefficient of variation (CV) of each subject categorized as continuous vs. bolus can be found in Table 2. The mean CV of both intervention days was 18.6% for continuous and 40% for bolus (Figure 1b). The CV of both groups differed significantly (*p* = 0.005). The visual representation of the glucose level progression over all measured time points at two study days individually is shown in Figure 1c and d.

### 2.3. DNA Damage and Oxidative Stress Markers

#### 2.3.1. DNA Damage

**Figure 2:**
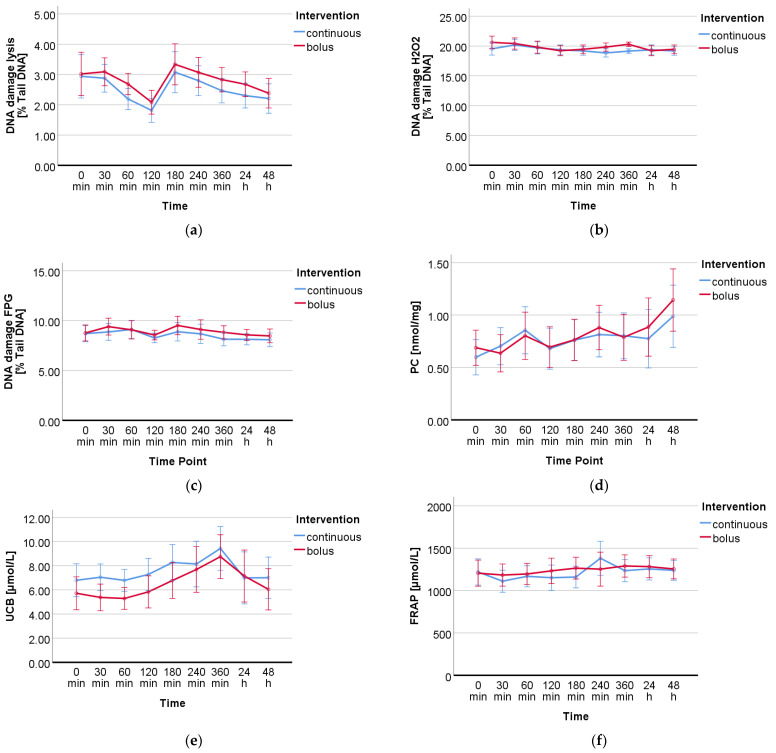
Progression of DNA damage and oxidative stress markers over the period of the interventions calculated by RM-ANOVA. DNA damage measured by comet assay with 3 different treatments: (**a**) no additional treatment, (**b**) treatment with H_2_O_2_, and (**c**) treatment with FPG. (**d**) Protein carbonyls, (**e**) unconjugated bilirubin, and (**f**) total antioxidant capacity by FRAP assay.

DNA damage measured by the standard treatment lysis resulted in a significant time effect (*p* < 0.001). Significant time effects (*p* = 0.001; *p* = 0.015) were also found for the specific treatments with the oxidant H_2_O_2_ and the lesion-specific enzyme FPG, respectively. However, there was no group or time-group effect in any of the three treatments. The mean values of all time points from both intervention groups (continuous vs. bolus) are presented in Table A2. The effects of the intervention (continuous vs. bolus) on DNA damage are shown in Table 3. Figure 2 shows the visual progression of DNA damage during treatment with lysis (a), H_2_O_2_ (b), and FPG (c).

#### 2.3.2. Oxidative Stress Markers

For the analyzed oxidative stress markers UCB and PC, a significant time effect was observed over the course of the study days (UCB *p* < 0.001, PC *p* < 0.004); FRAP showed only a tendency (*p* = 0.082). No group or time-group effect was found for any of the markers. The data (mean ± standard deviation) of all measured DNA damage and oxidative stress markers from both groups (continuous vs. bolus) are presented in Table A2. The effects of the intervention (bolus vs. continuous glucose administration) on all DNA damage and oxidative stress markers are summarized in Table 3. In Figure 2d–f, the progressions of the respective markers are shown visually.

## 3. Discussion

This study was the first to investigate the effects of GV on DNA damage as well as the oxidative stress markers PC, UCB, and FRAP in healthy male participants. GV was determined by CGM. The measured glucose levels showed a significant difference between the two groups, continuous and bolus, and over the period of the study (Figure 1a). Furthermore, the measured CV showed a significant difference (*p* = 0.005) between the two groups (Figure 1b). Looking in depth, Figure 1c,d show that the two glucose administrations display a clear picture over the period of 8 and 17 measurement time points, respectively. The main focus of this work was on the measurement of DNA damage in whole blood. The comet assay is a well-established method to determine single- and double-strand breaks in the DNA of various cell types [23,24,25] and has already been used to investigate the effects of type 2 diabetes [26,27]. Additional treatments using the oxidant H_2_O_2_ as well as restriction enzymes such as FPG give a more in-depth overview of the antioxidant status of the cells as well as on specific types of damage such as oxidized bases. A significant time effect was found in all three treatments; however, no significant group or time-group effect was found in any of them. To create a larger overall picture of the total oxidative status, antioxidants such as UCB and FRAP as well as oxidized byproducts as PC were determined. The determination of PC by 2,4-dinitrophenyl hydrazine is a standard method to detect protein oxidation in plasma [28]. UCB is a degradation product of the blood pigment hemoglobin and acts as an endogenous non-enzymatic antioxidant. There is evidence that bilirubin may serve as a biomarker for reduced chronic diseases, particularly cardiovascular disease, making it of high interest for the investigation of GV [29]. The determination of UCB as well as that of the antioxidative potential by FRAP are methods for the indirect determination of oxidative stress. The measured markers for this study have been used in numerous human studies for this purpose and are suitable to assess short- and long-term changes in the body [15,30,31]. We revealed a significant time effect for all measured DNA damage and oxidative stress markers, with the exception of FRAP, which showed only a tendency, over the period of the nine measured blood sampling points within 48 h. However, there was no significant difference between the two groups, continuous and bolus. The measured time effects raise questions about how they might occur. These could be explained by the circadian cycle of oxidative stress in the human body. An increasing amount of data indicates that the circadian regulation of protein expression is strongly involved in the organismic response to oxidative stress. Differences in DNA damage, lipid peroxidation, and protein oxidation at different times of the day have already been identified, and these variations are directly related to the circadian cycle of protective antioxidant molecules and enzymes. Whereas superoxide dismutase (SOD), glutathione peroxidase (GPx), glutathione reductase (GR), catalase (CAT), and uric acid are expressed the most in the morning, melatonin and ascorbic acid peak in the evening [32,33,34]. Variation in ROS levels due to circadian cycle has already been indirectly detected by the DNA damage marker 8-hydroxydeoxyguanosine (8-OHdG) as well as oxidative stress markers such as uric acid, malondialdehyde, or 8-isoprostane in blood or urine in healthy volunteers [35,36,37]. It is therefore reasonable to assume that the DNA damage and oxidative stress markers measured in the blood in this study may also vary as a result of the circadian cycle, but they are possibly also induced by glucose itself. The study had many strengths but also limitations. Particularly noteworthy are the strictly controlled conditions under which the study took place. The subjects were constantly monitored throughout the study, blood glucose values were assessed, and blood and plasma samples were taken at many time points. The number of participants was relatively small (*n* = 10); however, the crossover design, in which all participants received both treatments (continuous and bolus glucose administration), provided the necessary statistical power. Furthermore, only male participants took part in this study, as the female hormonal cycle could have interfered with the analyzed biomarkers. In addition, it is possible that higher glucose doses than three times 20 g and 60 g continuously could have triggered more severe changes. In the future, the number of participants could be enlarged, and women should also be considered, as should a group without glucose administration, to be able to distinguish between the circadian cycle and glucose-triggered effects. The investigation of different glucose levels in healthy volunteers was an important first step to better understand the effects of short-term changes in blood glucose levels and their clinical relevance. The results of the measured DNA damage and oxidative stress markers suggest that short-term changes in blood glucose levels do not have negative effects.

## 4. Materials and Methods

### 4.1. Participation Criteria and Study Design

**Figure 3:**
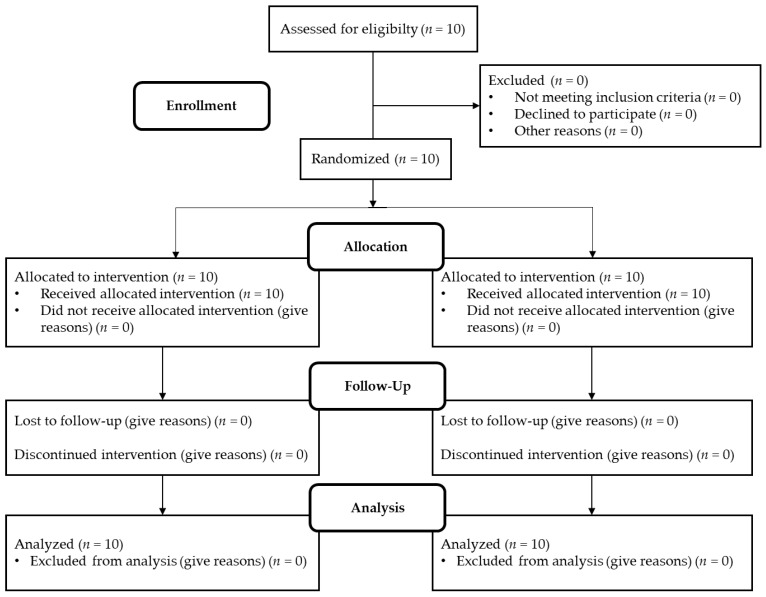
Flowchart of progress through the study phases.

The study took place in March 2019 at St. John of God Hospital in Linz, Austria. It was approved by the local joint research ethics committee of St. John of God Hospital Linz. This study investigated and described the effects of continuous vs. acute glucose administration on seven cardiovascular biomarkers (BMP6, SLAMF7, LOX-1, ADAMTS13, IL-1RA, IL-4RA, PTX3) [22]. The following inclusion criteria had to be fulfilled: male, non-smoker, between 18 and 40 years of age, HbA1c levels in normal ratio, and no history of type 2 diabetes. If these criteria were met, subjects were screened for health based on their history, a physical examination, and an electrocardiogram. Excluded were individuals with infectious diseases or who were being prescribed medication. The flowchart of the study is shown in Figure 3. Baseline characteristics of the subjects are described in Table 4. The study was conducted as a cross-over study and took place on two different days, 7–21 days apart from the second study day. The study was single-blinded, and the subjects were not informed on either day which treatment they would receive. At 8 am, after fasting and abstaining from alcohol and caffeine for 24 h, they received in random order intravenously either 3 times 20 g of glucose dissolved in 100 mL water over 5 min within one hour (at time points t0 min, t60 min, and t120 min) or they received 60 g of glucose dissolved in 300 mL of water continuously over 3 h (starting with time point t0 min). In addition to the glucose treatment, all subjects received a weight-maintenance diet that provided them with at least 200 g of carbohydrates. They were equipped with two catheters, one for glucose administration and the other to allow easy blood withdrawal at multiple time points. Blood was drawn at a total of 9 time points (t0 min, t30 min, t60 min, t120 min, t180 min, 240 min, t360 min, 24 h, 48 h). VACUETTE polyethylene terephthalate glycol blood collection tubes (Greiner Bio-One, Kremsmünster, Austria) were used to collect both whole blood and EDTA plasma, which was frozen at −80 °C until analysis. Blood glucose levels were measured on both days over the period of the study days. Heart rate, temperature, and blood pressure were monitored regularly during the study period.

### 4.2. Laboratory Analyses

#### 4.2.1. Comet Assay

The Comet assay was used to determine the DNA damage. With this single cell electrophoresis as described by Draxler et al., 2021 [31] it is possible to determine the percentage of DNA damage of a single cell in whole blood. An amount of 10 µL of each whole blood sample was mixed with 200 µL of 0.8% low melting agarose (Thermo Fisher, 16520050, Waltham, MA, USA), dissolved in phosphate-buffered saline (PBS) buffer (Merck, D8537, Rahway, NJ, USA), and applied as 5 µL spots on 4 microscopy slides coated with normal melting agarose (Thermo Fisher, 16550100). All slides are then incubated in a lysis solution (pH = 10) to dissolve the cell walls. After lysis, three slides from each sample were incubated for either 15 min in a 100 µM H_2_O_2_ solution and washed afterwards in PBS for 2 min or with the lesion-specific enzyme FPG (NEB, M0240L, Ipswich, MA, USA) or only the buffer in which FPG is dissolved (as a blank) for half an hour at 37 °C. After the treatment steps, all 4 slides were placed in an alkaline electrophoresis solution (pH = 13), and after 20 min of unwinding phase, electrophoresis ran for 30 min at 25 V, 150 W, and 300–350 mA. After 3 washing steps with PBS buffer and 70% then 100% ethanol, the slides were dried overnight and then stored at 2–8 °C until microscopic counting. Slides were stained with 0.03% Gel Red solution and manually counted using a fluorescence microscope (Nikon, Minato City, Tokyo, Japan) and “Comet Assay IV” counting software. Since single cells resemble comets closely under the microscope, results are reported as “% tail length” versus “% head length”. 

#### 4.2.2. Ferric Reducing Antioxidant Power (FRAP) Assay

The total antioxidants in the samples were determined by FRAP assay in EDTA plasma. The method was adapted from Benzie et al. (1996) [38]. An amount of 10 µL of plasma was mixed with 30 µL aqua dest. and 300 µL FRAP reagent (50 mL acetate buffer, 5 mL each of TPTZ solution and ferric chloride hexahydrate reagents) and applied to a 96- well microplate together with the concentration FeSO_4_ standards (100–2000 µM) (Merck) and a Trolox (Merck) control and measured at 540 nm after 6 min incubation at 37 °C. The antioxidant concentration [µmol FRAP/L] could be determined from the absorbance via the standard curve.

#### 4.2.3. Protein Carbonyls (PCs)

PCs were determined by indirect measurement using an albumin standard according to the adapted method of Levine et al. (1990) [39]. For each sample, 20 µL EDTA plasma was aliquoted into two different tubes on ice and mixed with 180 µL aqua dest. One tube was used to determine the protein content and the other to determine the PC content. The first tube was treated with 500 µL of 2 M hydrochloric acid (HCl) and the second with 500 µL of 0.2% 2,4-dinitrophenyl hydrazine (DNPH) (Merck, D199303) (in 2 M HCl). The carbonyl groups in the plasma were derivatized by DNPH to a yellow-colored dinitrophenyl hydrazine complex. After 15 min of incubation in the dark, both tubes were precipitated with 500 µL 20% TCA (Merck, 91230) and centrifuged for one minute at 1300× *g* and room temperature (23 °C). The supernatant was removed, and after 3 washing steps with 1 mL ethanol-ethyl acetate (1:2), samples were air-dried for 2 min and then mixed with 1 mL 6 M guanidine 500 mM KPO_4_ buffer and incubated in the dark for 15 min with occasional vortexing. The samples were then applied to a 96-well UV microplate and measured photometrically at 370 nm for protein carbonyl determination and at 276 nm for protein determination. The concentration of PC could be calculated indirectly based on the protein concentration of the samples and the albumin standard (0.5–3 mg/mL).

#### 4.2.4. Unconjugated Bilirubin (UCB)

UCB concentration in EDTA plasma was determined by high-performance liquid chromatography (HPLC) (Merck Hitachi LaChrom, Selm, Germany) as previously described by Wallner et al. (2012) [40]. A mobile phase consisting of 3.5% water and 96.5% methanol was used for the analysis. An amount of 50 µL of EDTA plasma was mixed with 200 µL mobile phase, then centrifuged for 10 min at a speed of 14,000 RPM and a temperature of 4 °C. An amount of 120 µL of the supernatant was pipetted into vials and placed in the autosampler. Analysis was performed using a Fortis C18 HPLC column (4.6 × 150 mm, 3 mm), a Phenomenex Security Guard cartridge for C18 HPLC columns (4 × 3 mm), and a photodiode array detector (PDA, Shimadzu, Tokyo, Japan). The bilirubin standard used was from Sigma Aldrich (St. Louis, MO, USA) (B4126).

### 4.3. Statistical Analysis

Statistical analyses were performed using Microsoft Excel 2019 and IBM SPSS Statistics 28 software. A repeated-measures analysis of variance (RM-ANOVA) was used for statistical evaluation of DNA damage and oxidative stress markers across the 9 time points and comparison of blood glucose levels of both groups. Greenhouse-Geisser correction was always applied when sphericity could not be assumed according to Mauchly. All time points were tested for normal distribution using Shapiro-Wilk tests. GV was measured by coefficient of variation (CV). CV was calculated for each participant across all measurement time points and both groups (standard deviation/mean) × 100 and expressed as a percentage. Moreover, CV was determined from all participants and divided into continuous and bolus groups. A Wilcoxon test was applied to compare the non-normally distributed CV values of both groups.

## 5. Conclusions

In conclusion, bolus vs. continuous intravenous administration of glucose did not significantly affect DNA damage in whole blood in any of the three treatments with lysis, H_2_O_2_, and FPG or oxidative stress measured with PC, UCB, and FRAP in plasma from healthy men. However, a significant time effect was observed for DNA damage, PC, and UCB, which could probably be explained by the circadian cycle of ROS response over the course of the day in the human body. This was the first study that provided insights into possible effects of short-term GV on the male healthy body. Even though hyperglycemia in the long-term results in increased ROS accumulation, no negative effects could be detected within a 48 h period based on the measured markers of DNA damage and oxidative stress. Further studies in a similar design with a larger and more diverse cohort could provide further evidence on the still relatively unknown phenomenon of GV.

## Figures and Tables

**Table 1:** Characteristics of the study participants.

	*n*	Minimum	Maximum	Mean	Standard Deviation
Age at participation	10	21	30	25	3
Pulse ECG (bpm)	10	53	88	62	11
Systolic blood pressure (mmHg)	10	107	138	120	10
Diastolic blood pressure (mmHg)	10	59	87	74	7
Size (cm)	10	174	190	180	5
Weight (kg)	10	73	100	83	9
BMI (kg/m^2^)	10	22.5	30.9	25.6	2.5
Waist circumference (cm)	10	81	110	90	8

**Table 2:** Coefficient of variation of the individual participants in comparison continuous vs. bolus in %.

Patient Code	CV Continuous (in %)	CV Bolus (in %)
A	9.65%	44.66%
B	11.85%	21.35%
C	16.22%	44.26%
D	26.48%	37.60%
E	18.42%	40.29%
G	27.12%	45.09%
H	10.81%	41.21%
I	27.64%	41.76%
K	12.25%	40.91%
M	25.49%	42.63%

**Table 3:** *p*-value of DNA damage and oxidative stress markers (time, time × group, and group effect).

Marker	Time Effect (*p*-Value)	Group Effect (*p*-Value)	Time × Group (*p*-Value)
Lysis [% tail intensity]	<0.001 *	0.301	0.829
H_2_O_2_ [% tail intensity]	0.001 *	0.377	0.059
FPG [% tail intensity]	0.015 *	0.341	0.728
PC [nmol/mg]	0.004 *	0.679	0.904
UCB [µmol/L]	<0.001 *	0.222	0.598
FRAP [µmol/L]	0.082	0.700	0.448

* Significant effects.

**Table 4:** Baseline characteristics of the participants.

Patient Code	Year of Birth	Age at Participation	Pulse ECG (bpm)	RR (mmHg)	Weight (kg)	BMI (kg/m^2^)	Waist Circumference (cm)	Body Size (cm)	HbA1c (mmol/mol)
B	1988	30	67	115/73	73	23.8	87.5	175	5.2
A	1997	22	54	133/75	100	30.9	110	180	5.5
K	1994	24	70	138/87	75	23.4	82.5	179	5.1
G	1992	26	55	115/71	79	24.9	88	178	5.2
C	1998	21	63	125/79	78.4	23.9	81	181	5.2
i	1996	23	53	107/76	78.5	25.9	92	174	5.3
E	1993	26	88	121/73	94	27.8	87	184	5.5
M	1993	26	54	124/74	86	27.1	90	178	4.9
D	1991	28	57	107/59	92.2	25.5	90	190	5.1
H	1993	27	57	116/68	77	22.5	90	185	5.1

## Data Availability

The data presented in this study are available on request from the corresponding author.

## Appendix A

**Table A1:** Blood glucose levels in mg/dL with continuous and bolus administration.

Time Point	*n*	Minimum	Maximum	Mean	Standard Deviation	Variance
Continuous 0 min	10	82	101	93	7	44
Continuous 30 min	10	106	156	135	16	240
Continuous 60 min	10	92	169	135	28	799
Continuous 120 min	10	100	142	121	15	226
Continuous 180 min	10	83	125	109	13	155
Continuous 360 min	10	83	93	88	4	13
Continuous 24 h	10	85	102	94	6	31
Continuous 48 h	10	85	101	93	5	23
Bolus 0 min	10	85	101	93	5	23
Bolus 5 min	9	153	236	196	32	1047
Bolus 10 min	9	212	308	246	31	987
Bolus 15 min	9	200	275	226	23	507
Bolus 30 min	10	117	193	153	25	638
Bolus 60 min	10	67	137	96	19	359
Bolus 65 min	8	126	247	172	38	1425
Bolus 70 min	8	161	288	223	39	1500
Bolus 75 min	8	169	296	216	42	1742
Bolus 120 min	10	55	131	83	20	416
Bolus 125 min	8	95	248	162	53	2785
Bolus 130 min	7	155	322	212	67	4517
Bolus 135 min	8	147	265	198	41	1698
Bolus 180 min	10	59	104	76	12	149
Bolus 360 min	10	78	92	85	5	22
Bolus 24 h	9	83	101	91	7	48
Bolus 48 h	10	77	107	93	9	86

**Table A2:** Measured markers at different time points (continuous vs. bolus). The values given are the mean ± standard deviation.

Time Point	DNA Damage Lysis [% Tail Intensity]	DNA Damage H_2_O_2_ [% Tail Intensity]	DNA Damage FPG [% Tail Intensity]	Protein Carbonyles [nmol/mg]	Unconjugated Bilirubin [µmol/L]	FRAP [µmol/L]
Continuous 0 min	2.94 ± 1.22	19.53 ± 0.79	8.70 ± 1.18	0.60 ± 0.25	6.79 ± 1.97	1219 ± 247
Bolus 0 min	3.02 ± 0.92	20.61 ± 2.08	8.76 ± 1.24	0.69 ± 0.25	5.71 ± 2.12	1205 ± 216
Continuous 30 min	2.88 ± 0.63	20.19 ± 1.68	8.86 ± 1.23	0.70 ± 0.14	7.04 ± 1.26	1110 ± 177
Bolus 30 min	3.09 ± 0.74	20.41 ± 1.11	9.39 ± 1.31	0.64 ± 0.35	5.37 ± 1.97	1183 ± 214
Continuous 60 min	2.19 ± 0.43	19.70 ± 1.38	9.11 ± 1.48	0.86 ± 0.39	6.78 ± 1.18	1168 ± 155
Bolus 60 min	2.68 ± 0.61	19.81 ± 1.68	9.07 ± 1.26	0.80 ± 0.28	5.28 ± 1.52	1195 ± 215
Continuous 120 min	1.81 ± 0.38	19.36 ± 1.19	8.26 ± 0.68	0.68 ± 0.27	7.27 ± 1.48	1152 ± 195
Bolus 120 min	2.08 ± 0.74	19.22 ± 1.32	8.55 ± 0.77	0.69 ± 0.31	5.83 ± 2.41	1233 ± 251
Continuous 180 min	3.08 ± 1.12	19.18 ± 0.92	8.88 ± 0.91	0.76 ± 0.33	8.27 ± 1.96	1160 ± 215
Bolus 180 min	3.34 ± 0.90	19.45 ± 1.25	9.51 ± 1.75	0.76 ± 0.26	6.77 ± 2.47	1264 ± 169
Continuous 240 min	2.80 ± 0.90	18.85 ± 1.12	8.67 ± 1.32	0.81 ± 0.40	8.14 ± 3.22	1382 ± 380
Bolus 240 min	3.07 ± 0.55	19.82 ± 0.95	9.10 ± 1.59	0.88 ± 0.22	7.68 ± 2.42	1253 ± 192
Continuous 360 min	2.46 ± 0.66	19.15 ± 0.64	8.13 ± 0.47	0.80 ± 0.36	9.42 ± 2.64	1235 ± 142
Bolus 360 min	2.83 ± 0.55	20.29 ± 0.49	8.82 ± 1.34	0.79 ± 0.30	8.74 ± 2.82	1290 ± 241
Continuous 24 h	2.30 ± 0.68	19.36 ± 1.19	8.12 ± 0.68	0.77 ± 0.35	6.99 ± 3.07	1255 ± 207
Bolus 24 h	2.68 ± 0.54	19.22 ± 1.32	8.56 ± 0.98	0.89 ± 0.48	7.14 ± 3.40	1282 ± 184
Continuous 48 h	2.21 ± 0.78	19.18 ± 0.92	8.07 ± 0.76	0.99 ± 0.31	7.00 ± 2.15	1238 ± 181
Bolus 48 h	2.38 ± 0.68	19.45 ± 1.25	8.46 ± 1.26	1.14 ± 0.55	6.05 ± 2.93	1255 ± 172
